# Patients’ Experiences of Using Smartphone Apps to Support Self-Management and Improve Medication Adherence in Hypertension: Qualitative Study

**DOI:** 10.2196/17470

**Published:** 2020-10-28

**Authors:** Ciara M McBride, Eimear C Morrissey, Gerard J Molloy

**Affiliations:** 1 School of Psychology National Univeristy of Ireland Galway Galway Ireland; 2 Health Behaviour Change Research Group National Univiersity of Ireland Galway Galway Ireland; 3 Medication Adherence Across the Lifespan Research Group National University of Ireland Galway Galway Ireland

**Keywords:** hypertension, self-management, mobile applications, feasibility, usability, medication adherence, qualitative research, digital technology, mobile phone

## Abstract

**Background:**

Worldwide, hypertension control rates remain suboptimal despite clinically effective antihypertensive drug therapy. Patient failure to take medication as prescribed (ie, nonadherence) is the most important factor contributing to poor control. Smartphone apps can facilitate the delivery of evidence-based behavior change techniques to improve adherence and may provide a scalable, usable, and feasible method to deliver self-management support.

**Objective:**

The aim of this study is to explore patients’ experiences of the usability and feasibility of smartphone apps to support self-management and improve medication adherence in hypertension.

**Methods:**

A qualitative descriptive study was conducted. A total of 11 people living with hypertension from the West of Ireland were sampled purposively and interviewed about their experience of using a self-management app for a 4-week period, which included two key functionalities: self-monitoring of blood pressure (BP) and medication reminders. Thematic analysis was carried out on the semistructured interview data.

**Results:**

Participants’ age ranged from 43 to 74 years (mean 62 years, SD 9.13). Three themes were identified: digital empowerment of self-management, human versus digital systems, and digital sustainability. Although patients’ experience of using the technology to self-monitor BP was one of empowerment, characterized by an enhanced insight and understanding into their condition, control, and personal responsibility, the reminder function was only feasible for patients who reported unintentional nonadherence to treatment. Patients experienced the app as a sustainable tool to support self-management and found it easy to use, including those with limited technological competence.

**Conclusions:**

The study’s findings provide new insights into the experience of using apps to support medication adherence in hypertension. Overall, the data support apps as a usable and feasible method to aid self-management of hypertension and highlight the need for personalized functionality, particularly with regard to medication adherence reminder strategies. The study’s findings challenge the perspective that the use of these technologies to support self-management can inevitably add to the burden of treatment experienced by patients.

## Introduction

Hypertension has been identified as the leading modifiable risk factor for cardiovascular disease and consequently represents a major cause of premature morbidity and mortality due to adverse cardiovascular and cerebrovascular events [1]. Blood pressure (BP) control and management of hypertension can be achieved through antihypertensive drug treatment, which has proved to be clinically effective [2,3]. However, recent evidence (including control rates across 12 countries) suggests that BP control through antihypertensive treatment is suboptimal, with at least 20% of those prescribed treatment failing to achieve control [4]. Findings from nationally representative community-based studies, such as The Irish Longitudinal Study on Ageing, report control rates as low as 50% [5]. Patient nonadherence (ie, failure to take medication as prescribed) continues to be recognized as the most critical variable in suboptimal BP control through antihypertensive treatment [6].

Some intervention approaches to support self-management of hypertension and enhance adherence have been shown to be effective in improving BP control [7] and increasing adherence [8]. These interventions have included a heterogeneous range of behavior change techniques to improve medication adherence [9]. Systematic and meta-analytic review evidence supports self-monitoring of BP as a successful intervention component to reduce mean systolic and diastolic BP readings [10,11] and increase adherence to antihypertensive therapy [12]. Automated medication reminders (ie, environmental prompts or cues) offer a simple, yet empirically established intervention component associated with enhanced adherence and improved BP control among high-risk patients with hypertension [13]. It is clear that such active intervention ingredients can be delivered using innovative digital technologies, including smartphone apps, which might constitute a scalable, potentially usable, and feasible method to deliver self-management support to patients with hypertension [14,15].

Despite the potential of such devices to improve adherence, a contemporary theoretical perspective proposes that newer technologies that encourage a proactive approach to patient care and can be easily shifted from the clinic to the community may serve to place new demands on patients, causing them to experience an increased burden of treatment [16]. Furthermore, an exploration of patients’ perspectives on the possible adoption of smartphone apps to support self-management and improve adherence in hypertension has revealed important concerns, including the potential of such technologies to increase health-related anxiety, and the authors have cautioned that the current patient perspective might be best characterized as one of *ambivalence* [15]. An exploration of the patient experience of using these technologies in an ongoing community context is now warranted to provide timely insights into these concerns. Therefore, the aim of this study is to explore patients’ experiences of the usability and feasibility of smartphone apps to support self-management and improve medication adherence in hypertension.

## Methods

### Design

A qualitative descriptive study was conducted. The Consolidated Criteria for Reporting Qualitative Research Checklist was used to ensure explicit reporting on how this study was carried out (Multimedia Appendix 1) [17].

### Participants and Recruitment

Participants were recruited through Croí (the West of Ireland Cardiac Foundation), a heart and stroke charity located in Galway city. Recruitment emails advertising the study were sent to database records of individuals who had used the charity’s service until March 2019. Interested patients living with hypertension contacted the primary researcher by telephone or email to obtain further study information and ensure eligibility. To be eligible for participation, patients must have been prescribed at least one form of antihypertensive therapy and own an Android smartphone. Purposive sampling was used to achieve adequate variability in age, sex, and complexity of the medication regime.

### The App

The smartphone app used is called *BP Journal*. BP Journal is commercially available (Google Play Store), serves as a companion app to a clinically validated home BP monitor, and was chosen by the research team as it is typical of medication management apps for hypertension in that it includes two key functionalities: self-monitoring of BP and medication reminders. Users of the app create a personal profile and set daily reminders to take BP medication and readings. The app allows for the self-reporting and storage of BP readings, producing feedback of BP measurements in the form of statistics and interactive charts. An export function provides users with an opportunity to print or send via email BP data in comma-separated values or PDF format.

### Procedure

Participants attended an initial session during which they downloaded the app and created their personal profile. They were then provided with a home BP monitor (A&D Medical model UA-767S-W) and shown how to navigate the equipment. Participants were requested to use these materials to support hypertension self-management for 4-weeks. To ensure participants became familiar with app functionalities, it was recommended that they took BP readings at least twice per week (unless they had a pre-established self-monitoring routine). The 1-month feasibility duration was based on previous mobile health feasibility studies using 2- to 4-week periods [18,19].

Semistructured, face-to-face interviews were facilitated and audio-recorded by the primary researcher (CM, a female MSc graduate in health psychology with experience and training in qualitative research methods of both collection and analysis) to elicit patients’ experiences of using the app. The interview topic guide, which was centered around questions relating to feasibility and usability, was composed by reviewing qualitative research in the area and revised during the data collection process to be responsive to unforeseen issues. Therefore, an iterative approach was adopted to ensure that the researcher did not restrict the analysis to only issues anticipated as relevant [20]. Feasibility was defined in terms of practicability of using the app to self-manage hypertension and the extent to which participants successfully (or unsuccessfully) used the BP Journal app for the 4-week period (Multimedia Appendix 2). The interviewer had no previous relationship with participants before the study commenced, and the duration of the interviews ranged from 9 to 29 min. All data were collected at Croí House, the charity’s dedicated facility. No nonparticipants were present during the data collection process other than the primary researcher.

The System Usability Scale (SUS) [21] was distributed to participants before the commencement of interviews to provide descriptive data on the perceived usability of the app. Scores on the SUS range from 0 to 100, with higher scores indicating greater perceived *ease of use*. A score of 68 or above is considered above average [22].

### Data Analysis

Interviews were transcribed verbatim and analyzed using thematic analysis, following the six phases proposed by Braun and Clarke [23]. “Thematic analysis is a method for identifying, analyzing, and reporting patterns (themes) within data” and is celebrated for its accessible and theoretically flexible approach to analyzing qualitative data. A realist inductive approach, a data-driven approach to analysis that reports on the experiences of participants without trying to fit into an existing coding frame or follow prior theoretical conceptions, was taken [23]. During phase one, the familiarization process involved active reading of the transcripts. Phase two involved the generation of an initial list of codes and involved the assignment of sections of the transcripts to descriptive codes. Phase three involved refocusing the analysis by sorting codes into potential themes. This involved the creation of preliminary categories comprising codes that were conceptually linked. Phase four included refining a set of candidate themes produced during phase three, and phase five involved defining and naming the themes. Phase six included the write-up of the analysis in a concise, coherent, and logical manner [23]. The refinement of themes during phase five resulted in the organization of patterns within each theme into subthemes, which also enhanced the coherence and presentation of the results. In an effort to enhance reflexivity, 2 members of the research team (2 health psychologists) joined the primary researcher to review preliminary categories, refine candidate themes and subthemes, and each contributed to the analysis [24]. NVivo version 12 (QSR International) was used to facilitate the analysis.

### Ethical Approval

Ethical approval was sought and granted for this study by the School of Psychology Research Ethics Committee at the National University of Ireland, Galway.

## Results

### Participant Characteristics

Patient characteristics are summarized in Table 1. Three themes were identified in the data: digital empowerment of self-management, human versus digital systems, and digital sustainability. To reflect patients’ perceived usability of the technology, the SUS score of each participant complements supporting quotations below.

**Table 1:** Patient characteristics (N=11).

Characteristics				Values
**Demographic characteristics**				
	Age (years), mean (range)		62 (43-74)	
	Female, n (%)		6 (54)	
	Urban, n (%)		8 (73)	
	Married, n (%)		9 (82)	
	Single, n (%)		2 (18)	
	Working full-time, n (%)		4 (36)	
	Working part-time, n (%)		1 (9)	
	Unemployed, n (%)		1 (9)	
	Retired, n (%)		5 (46)	
	Private health insurance, n (%)		5 (46)	
**Clinical characteristics**				
	Number of antihypertensive medications, mean (range)		1.73 (1-3)	
	>12 months since diagnosis, n (%)		11 (100)	
	Presence of multimorbid conditions, n (%)		9 (82)	
**Other**				
	Previous use of a smartphone to manage hypertension, n (%)		1 (9)	
	SUS^a^ score, mean (range)		89.1 (70-97.5)	
	**Self-rated technology literacy, n (%)**			
		High	7 (64)	
		Medium	4 (36)	

^a^SUS: System Usability Scale.

### Digital Empowerment of Self-Management

The theme of digital empowerment of self-management refers to patients’ experiences of using the technology to self-monitor BP and is represented by two subthemes: *insight and understanding* and *control and responsibility* Although none of the participants had a regular self-monitoring routine at the time of study commencement, some reported prior occasional use of monitoring equipment. For others, the study provided their first experience of self-monitoring BP. Many patients who reported prior use of monitoring devices described how using the app had provided them with a better monitoring system, when compared with previous *ad hoc* systems:

The thing about [using the app] is that it is more regular...up to this I would only use the monitor when I felt I had high BP...doing it on regular basis is a better monitoring system, whereas mine was ad hoc.

Male, 65 years; SUS 87.5

#### Insight and Understanding

Several patients valued the output information produced by the self-monitoring function and felt it provided them with a greater insight into the state of their BP. The retrieval of visual feedback in the charts offered to participants more meaningful information and a feeling of empowerment:

The good thing about the app was I could see it visually; visual things mean a lot more to me.

Male, 65 years; SUS 87.5

All the information it gave me about the state of the BP [was the most helpful aspect of the app]...I could see the different measurements myself and see the charts up and down.

Female, 63 years; SUS 97.5

Many participants expressed how visualizing BP data in the charts made their condition more tangible, whereas the visual qualities in the app helped others make sense of and interpret BP readings. Some acknowledged that using the self-monitoring function had provided them with an enhanced understanding of the asymptomatic nature of hypertension and the hazards of judging their condition by how they felt, thereby reconstructing the way they perceived their condition:

You are getting an accurate account of what’s happening in your body...normally you are judging yourself by how you feel...I was surprised my BP was higher than I thought...that made me aware sometimes I might feel good and be busy, but I was overlooking.

Male, 65 years; SUS 87.5

Visualizing fluctuations in BP readings was perceived as interesting by several and sparked curiosity. Fluctuations in readings despite feelings of accurate medication-taking behaviors evoked a sense of concern in some patients regarding whether their medication was working to control their BP. For others, the retrieval of consistent readings verified that their BP was under control:

It was a nice app...it told me my BP was fluctuating even though it was the same time of day. I am considering going back to my doctor and showing him the results on my follow-up...there might be another tablet that might be better.

Female, 71 years; SUS 97.5

#### Control and Responsibility

Many patients expressed how their newly established self-monitoring regime had provided them with an improved feeling of control over their condition between consultations in primary care. They described how this had offered them reassurance and had promoted an increased feeling of responsibility for one’s health. One participant described how she thought this improved feeling of control might function to decrease health anxiety in patients who were worried about their BP:

When you go and get your BP taken and you’re put on medication if everything is going as it should you may not go back to the doctor, you don’t know what’s going on...six months can make a big change. I just heard of another person, they are panicked about their BP and ringing the doctor...if you had this at home you might calm down...you have this back up to know that everything is controlled...you’re looking after yourself and have your own check.

Female, 50 years; SUS 95

Several of the participants described how their improved feeling of responsibility would help them *take action* if their BP readings were consistently out of range. Many patients for whom the study provided them with their first experience of self-monitoring BP discussed how their new sense of responsibility was stronger than what they had felt with the conventional standard office monitoring system in primary care:

If there were any unusual stuff happening, I’d pick it up...sometimes you go to a GP to get a check-up, but you wouldn’t pick it up there and then...if you are doing this self-checking yourself...if there is something unusual happening you could take action.

Male, 56 years; SUS 92.5

Feeling empowered, some reported instances during which the app had encouraged them to play an active role in consultations. This occurred when patients used the app to guide conversation with their general practitioner (GP). A few patients underwent ambulatory monitoring, followed by the detection of uncontrolled BP and, in turn, experienced changes to their medication:

I showed it to my doctor, and she did a blood pressure monitor...[It] advised that my blood pressure was very high...I feel glad I found it out.

Female, 63 years; SUS 97.5

### Human Versus Digital Systems

The theme of *human versus digital systems* focuses on patients’ experiences of the technology to support reminder strategies in daily self-management and is represented by two subthemes: *utility of the digital system* and *interference of the digital system.*

#### Utility of the Digital System

For those patients who reported they might frequently forget to take medication (ie, unintentional nonadherence) and could recognize their current medication-taking system was not perfect, the medication reminder was experienced as useful:

Some mornings I was rushing and only for it did beep I probably would have forgotten to take them...it’s a tool, it helps because we can forget easily.

Female, 50 years; SUS 95

I mean the reminder was good because it told you at the time to take your medication and there is a chance with me, I would forget...there was a couple of times I totally forgot.

Male, 74 years; SUS 70

One participant described how the medication reminder was becoming integrated into his everyday routine:

The reminder was excellent...I’d look at the clock and say it wasn’t some message coming in...I knew it was time to take my medication.

Male, 65 years; SUS 87.5

#### Interference of the Digital System

The medication reminder was experienced as less practical among patients who expressed a strong satisfaction with their current *human system*, linking medication taking to events in their daily routines. These patients varied in future intent to engage with the reminder. Although several patients could see the benefit of using the reminder if they were out of routine, others considered the reminder as unnecessary:

I didn’t need reminding about my tablets in all the years I’m on them I’ve probably forgotten to take them twice ever...it’s part of my routine after breakfast and before I go to bed...having said that I am going on holidays in a few weeks, it will be good for that.

Female, 43 years; SUS 87.5

One patient described how she had tried to stick to the time-based reminder but reverted to *old habits* out of fear that she might forget to take her medication. In this way, the *digital system* interfered with the patients pre-established medication routine. This patient expressed how she thought the reminder would be more feasible for patients who did not have strong habits:

I thought the reminder was good...if I was somebody starting off that hadn’t made all these habits...but for me after 25 or 30 years I couldn’t do it, I think it’s just old habits die hard.

Female, 63 years; SUS 97.5

### Digital Sustainability

The final theme to be identified was *digital sustainability.* The theme of digital sustainability refers to participants’ thoughts and ideas about the use of the technology in the future to support hypertension self-management and suggestions to improve app functionality. This theme is further represented by two subthemes: *long-term use of the digital technology* and *improvement suggestions*.

#### Long-Term Use of the Digital Technology

Most participants reported no concerns about using the technology going forward. Several reported that the app and monitor were easy to use despite limited technological competence:

Technology over the last couple of years...what I could do before I have kind of forgotten. I mean I [still] found the app very...once you did one or two testings with it, it became automatic.

Male, 74 years; SUS 70

Most participants expressed that they intended to continue using the technology after the study. The self-monitoring function was a major motivation for continued use. Many patients who did not own a monitoring device acknowledged that they intended to buy one. Perceiving the app as being beneficial in consultations encouraged sustainability:

I am actually going to buy a monitor of my own and if the app stays there I am going to continue taking readings...I will have it there to show the trend in my blood pressure, so I can pass it on to a doctor.

Male, 63 years; SUS 92.5

#### Improvement Suggestions

Important improvement suggestions included making the reminder more intrusive by adding an audio signal or a *snooze* function. Another suggestion included making the reminder function more comprehensive by allowing patients to confirm in the app that they had taken their medications. The utility for more comprehensive app functionalities, including components to promote adherence to subsequent lifestyle behaviors such as physical activity and diet, was also mentioned. Suggestions to increase the complexity of the app were weighed against a strong emphasis to keep app functionalities simple to ensure that the app was suitable for all users, particularly those who might not be savvy:

I find in life, it’s called the KISS principle...keep it straight and simple...and the app in my opinion is easy to use...you have to make it easy to use...remember your clients that you are giving it to are not savvy...

Male, 63 years; SUS 92

## Discussion

### Principal Findings

The data from these interviews provide new insights into patients’ experiences of the usability and feasibility of smartphone apps to improve adherence to hypertension. The patient experience of using the technology to support self-monitoring of BP was one of empowerment, characterized by an enhanced insight and understanding of hypertension, an improved feeling of control over health, reassurance, and increased patient responsibility. Patients’ experiences of using the technology to support reminder strategies were more varied; although the reminder function was useful for patients who reported unintentional nonadherence to treatment, it was less practical for those who reported existing context-based medication-taking habits. Overall, most patients experienced the technology as sustainable, reporting that they intended to continue to engage with the app in the future. Patients were confident in their use of the technology by the end of the feasibility period despite limited digital competence. This was complemented by descriptive data, which demonstrated that all participants scored above average on the SUS, a robust evaluation tool of perceived *ease of use* [25].

### Strengths and Limitations

This study provides timely data on the use of technology to support self-management of hypertension and to the researcher’s knowledge is the first to use qualitative methods to examine patients’ experiences of the usability and feasibility of smartphone apps specifically for medication adherence in hypertension. This extends research in this area [15] that focused on initial impressions of smartphone app use rather than using them over an extended period. This provides the perspective of users beyond an early novelty phase and provides new findings that have enhanced ecological validity. Semistructured, face-to-face interviews were appropriate because they prevented the loss of contextual and nonverbal data, allowing the interviewer to pick up on visual cues, such as how to proceed with dialogue [26]. In addition, individual interviews were appropriate as they allowed patients to express their own individual opinion, avoiding the social desirability bias, which might concern group-based methods of data collection. It is also likely that the research team members with different levels of expertise coming together to each contribute to the analysis heightened reflexivity, which is another potential methodological strength.

One limitation is the potential of sampling bias. Although purposive sampling was used, the sample was from a single geographic location and limited to Android smartphone users. It is also possible that this sample captures a more positive patient experience of self-management technologies, as it is based only on a subsample of patients who volunteered to participate and who had prior involvement with Croí, the Irish heart and stroke charity. This is due to the potential likelihood of the participants reflecting a more motivated, technologically inclined, and adherent group. It must also be recognized that all the patients had a hypertension diagnosis of >12 months, which could have influenced the findings.

### Comparison With Prior Research

Our findings support previous research investigating patients’ experiences of self-management technologies across other illness contexts. A metaethnography on digital interventions to support self-management covering a range of conditions (eg, asthma, chronic obstructive pulmonary disease, diabetes) has found that patients monitoring their health felt reassured by the insight provided and felt they had more meaningful conversations with health care practitioners [27]. The review also found that patients who could understand self-monitored data in the context of lifestyle behaviors (eg, medication adherence) felt an improved sense of control over their condition, which allowed them to find meaning in physiological self-monitored data. This is reflected in our theme of *digital empowerment of self-management* wherein patients expressed a sense of reassurance, played a more active role in consultations (or believed self-monitored data would allow them to do so), and ascribed meaning to visual feedback. Similarly, many patients linked readings to medication-taking behavior and felt improved control over their BP. Our theme of *digital empowerment of self-management* is also consistent with the findings of a thematic synthesis by Fletcher et al [28], which focused specifically on self-monitoring of BP in hypertension and found that increased control, autonomy, and self-efficacy enabled patients to move from a passive to an active role in consultations, promoting patient involvement in care.

Our findings support previous literature from a number of areas including information visualization, human-computer interaction, and medicine, which argues that making health data visible creates opportunities for patients to make sense of their illness, which in turn may lead to more effective self-management [29]. In our data, visualizing self-reported BP data made hypertension more tangible for patients and reconstructed the way they perceived their condition. These findings are consistent with the findings of a study by Hallberg et al [30], who found that using a mobile phone–based self-management system that included visual feedback of self-monitored BP data caused patients with hypertension to experience changes to illness perceptions, including timeline and treatment beliefs. These theoretical constructs from the Common-Sense Self-Regulation Model (CS-SRM [31]) have been consistently shown to predict adherence to antihypertensive therapy in quantitative studies [32,33]. These findings have also been supported in qualitative studies [34].

The findings of this study also lend support to emerging research that points to the possible utility of digital technology, especially smartphone apps, to support reminder strategies in some patients who report unintentional nonadherence to treatments (eg, [35]). This is reflected in our theme *human versus digital systems* wherein patients who reported unintentional nonadherence to antihypertensive medication found the digital reminder useful, whereas those who reported contextual-based habits did not.

### Implications for Practice, Research, and Design

Ineffective communication with patients with hypertension is correlated with poor adherence to antihypertensive therapy, and effective communication has been identified as an important obstacle for practitioners, largely due to a lack of understanding of hypertension in patients [36]. In our data, use of the technology enhanced patients’ understanding of hypertension and many reported uses of the app in consultations to facilitate discussion. The use of the app in consultations helped to engage patients in shared decision making regarding treatment. These findings suggest that the use of the technology can promote more effective interaction in practice, potentially inadvertently increasing adherence and patient involvement in care, and helping to bridge a gap in the traditional doctor-patient relationship. Despite this implication of this finding for practice, previous inquiries have shown that this shift in power balance is not always viewed as a positive thing by practitioners [37,38]. Another important consideration highlighted by a recent study on GPs’ perspectives that must be addressed in relation to the doctor-patient relationship and such technologies in practice concerns the issue of who holds the responsibility for self-monitored BP data [37].

The findings of this study have important implications for optimizing technological design. In addition to considering the feasibility of the suggestions made by these patients to promote the sustainability of the app, the authors recommend that future developers of adherence technologies for hypertension design contextual-based reminder functionalities to support habit development in patients who report unintentional nonadherence. Prospective memory is an important component of medication adherence and is supported by context-based cues more effectively than time-based cues [39]. This is reflected in our findings, as patients who were satisfied with their *human system* linked medication taking to events in their everyday lives. The potential capacity of such devices to support habit formation in hypertension may represent a key mechanism whereby adherence can be enhanced, as the extended CS-SRM has emphasized that habit strength is a significant theoretical predictor of unintentional nonadherence, and related evidence indicates that it may be the most potent predictor of long-term adherence to antihypertensive therapy [40].

Finally, it is noteworthy that a large proportion of participants who were living with hypertension in this study reported the presence of multimorbidity (defined as the presence of two or more chronic health conditions coexisting in one individual). Epidemiologic evidence suggests that multimorbidity is now the norm, rather than the exception [41]. Although the prevalence of multimorbidity in the study sample is higher than that reported in other studies, including community-based samples of patients with hypertension [42], the high prevalence reflects a need for medication management technologies to take such findings into account. Although the current focus was on hypertension self-management, it remains undetermined whether patients’ experience of self-managing hypertension in this study had an impact on their management of coexisting conditions. It is a potential avenue for future research to develop scalable methods to support the management of polypharmacy (use of multiple medications) to promote adherence in patients with multimorbidity. The authors are aware that preliminary research efforts are being made in this area (eg, [43]). However, a recent Cochrane review [44] examining interventions to manage multiple medications in older adults included limited studies that currently use digital technology to support self-management of multimorbidity. Careful development is required, which integrates behavior change science.

### Conclusions

The patients in this qualitative study experienced the technology as a usable and feasible method to support self-monitoring of BP. However, to experience the technology as feasible to support reminder strategies, this study’s findings suggest that the patients must first have found a need that their current medication-taking systems could not fill. The technology was generally experienced by these patients as a sustainable tool to support hypertension self-management in the long term. Overall, the data support apps as a usable and feasible method to support self-management and highlight the need for personalized functionality in relation to medication adherence reminder strategies. This study adds to a growing body of literature that challenges the perspective that the use of such self-management technologies will inevitably add to the patient burden of treatment [16].

## Appendix

Multimedia Appendix 1 Consolidated Criteria for Reporting Qualitative Research Checklist.

Multimedia Appendix 2 Interview Topic Guide.

## Abbreviations

BP: blood pressure
CS-SRM: Common-Sense Self-Regulation Model
GP: general practitioner
SUS: System Usability Scale
