# Supplementary material for: Patients’ Experiences of Using Smartphone Apps to Support Self-Management and Improve Medication Adherence in Hypertension: Qualitative Study
Source: JMIR Mhealth Uhealth. 2020 Oct 28;8(10):e17470. doi: 10.2196/17470 (PMC7657730; doi:10.2196/17470)
Supplement: Multimedia Appendix 1 [file mhealth_v8i10e17470_app1.pdf]

Item	Description
**Domain 1: Research team and reflexivity**	
Personal Characteristics	
1. Interviewer/facilitator	All interviews were conducted by CMB
2. Credentials	BSc, MSc
3. Occupation	MSc graduate in health psychology/researcher
4. Gender	Female
5. Experience and training	Experience and training in qualitative research methods of both data collection and analysis
Relationship with Participants	
6. Relationship established	The primary researcher had no relationship with participants before the study commenced
7. Participant knowledge of the interviewer	Participants had no knowledge of the interviewer except that she was a MSc student in health psychology
8. Interviewer characteristics	As the primary researcher was fully engaged in the research process it was difficult to avoid personal bias

Domain 2: Study design

Theoretical framework

9. Methodological orientation and theory	Thematic analysis was the methodological orientation underpinning the present study. Data was analysed using the six phases of reflexive thematic analysis outlined in Braun and Clarke [23]. A realist inductive approach, a data-driven approach to analysis which reports on the experiences of participants without trying to fit into an existing coding frame or follow prior theoretical conceptions was taken
Participant selection	
10. Sampling	Patients with hypertension living in the West of Ireland were recruited using purposive sampling to ensure adequate variation across a range of characteristics (eg, age, sex, complexity of medication regime)
11. Method of approach	In March 2019, participants were contacted through recruitment emails advertising the study that were sent to database records of service users of Croí, the West of Ireland Cardiac Foundation. Interested and eligible participants contacted the primary researcher by telephone or email to ensure eligibility and arrange the initial session
12. Sample size	A total of eleven hypertensive patients took part in the study
13. Non-participation	No participants refused to partake or dropped out of the present study
Setting	
14. Setting of data collection	All data was collected at Croí House, a dedicated heart and stroke facility located in Galway city, Ireland

15. Presence of non-participants	No non-participants were present during data collection, other than the interviewer (CMB)
16. Description of sample	An overview of patient characteristics is presented in Table 1
Data collection	
17. Interview guide	The interview topic guide was based on previous qualitative research in the area that used focus groups [15] and was refined and reviewed by the research team to suit the present research topic. The topic guide was revised during the data collection process in line with best practice guidelines, therefore, an iterative approach was taken
18. Repeat interviews	No repeat interviews were conducted
19. Audio/visual recording	Interviews were audio recorded
20. Field notes	Field notes were made before and after the interviews
21. Duration	The duration of interviews ranged from 9 minutes to 29 minutes
22. Data saturation	Data saturation was not used to determine the sample size as this is a problematic assumption of Braun and Clarkes [23] method of thematic analysis
23. Transcripts returned	Transcripts were not returned to participants for comment or correction

Domain 3: Analysis and findings

Data analysis

24. Number of data coders	Coding was carried out by the primary researcher (CMB) and partially by another member of the research team (EM). Inter-rater reliability was not calculated as this is a problematic assumption of Braun and Clarkes [24] method of thematic analysis
25. Description of the coding tree	The initial coding process during phase two took the form of open coding and used a data-driven approach. This occurred after familiarisation with the data and involved the assignment of sections of the transcripts to descriptive codes. The initial codes were collapsed under different categories, which were then refined into themes during phases three and four
26. Derivation of themes	Themes were identified from the data using an inductive approach
27. Software	NVivo version 12 was used to help facilitate the analysis
28. Participant checking	No feedback was obtained from participants regarding the findings of the study

Reporting

29. Quotations presented	Quotations have been presented to illustrate and support the findings. Quotations are accompanied by the gender, age and SUS score of the participants

30. Data and findings consistent	There is consistency between the data presented and the findings. The unit of analysis was the theme, rather than the frequency of statements
31. Clarity of major themes	The codes identified in the initial coding process were discussed in the larger research team. All of the major recurring themes are described in the findings
32. Clarity of minor themes	The subthemes are also described underneath each major theme in the findings
