# Supplementary material for: Patients’ Experiences of Using Smartphone Apps to Support Self-Management and Improve Medication Adherence in Hypertension: Qualitative Study
Source: JMIR Mhealth Uhealth. 2020 Oct 28;8(10):e17470. doi: 10.2196/17470 (PMC7657730; doi:10.2196/17470)
Supplement: Multimedia Appendix 2 [file mhealth_v8i10e17470_app2.pdf]

Multimedia Appendix

Interview topic guide.

Topic	Question	Probe
Opening	Why did you decide to take part in this study?<br>How long have you been diagnosed with high blood pressure/been taking anti-hypertensive medication?<br>What do you do yourself to manage your blood pressure (before study)?	
Previous use of digital technology	Before this study, had you used technology such as mobile phones, the internet, Apps in your everyday life (example: banking, twitter)?	Do you use such technologies in your everyday life for managing your health (examples: physical activity tracking, medication reminder, diet tracking and advice)?<br><br>Do you use such technologies useful and if so for what?
Feasibility	How did you find working with the blood pressure App in your everyday life over the past 4 weeks?	What did you like?<br>What did you dislike?<br>What did you find useful?<br>What did you think of the reminder?<br>What did you think about the output graphs?
	How did you find working with the monitor?	What did you like?<br>What did you dislike?
	Do you have concerns or difficulties in your use of the blood pressure App and blood pressure monitor in your everyday life?<br>How did using the App and monitor fit into your everyday routine?	
Suggested Improvements	Is there anything else that you think would be helpful or motivating to you in managing your hypertension?	Are there any specific improvements that could be made to the app (e.g., visuals, graphs)?

		What specific improvements would you like to see to the app to improve its benefits?<br>Are there any aspects of the blood pressure App and blood pressure monitor that you would definitely not be interested in using?
Usability	Why do you think such a tool might be useful to you?<br><br>How do you think the app looked?<br><br>Was the app easy or hard to use?<br><br>How did you find seeing your blood pressure readings on graphs?	What would you change to make it easier to use?
Closing	Would you use an App like this in the future?<br>Is there anything else about the app that you would like to discuss?	
